# A Novel Variable Selection Method Based on Binning-Normalized Mutual Information for Multivariate Calibration

**DOI:** 10.3390/molecules28155672

**Published:** 2023-07-26

**Authors:** Liang Zhong, Ruiqi Huang, Lele Gao, Jianan Yue, Bing Zhao, Lei Nie, Lian Li, Aoli Wu, Kefan Zhang, Zhaoqing Meng, Guiyun Cao, Hui Zhang, Hengchang Zang

**Affiliations:** 1 NMPA Key Laboratory for Technology Research and Evaluation of Drug Products, School of Pharmaceutical Sciences, Cheeloo College of Medicine, Shandong University, Jinan 250012, China; zlwolf96@163.com (L.Z.); huangruiqi1998@163.com (R.H.); gaolele1995@163.com (L.G.); y161600@126.com (J.Y.); zhaobing911@163.com (B.Z.); nielei2004@sdu.edu.cn (L.N.); lilian@sdu.edu.cn (L.L.); wual@sdu.edu.cn (A.W.); zkf050314@163.com (K.Z.); 2 Shandong Hongjitang Pharmaceutical Group Co. Ltd., Jinan 250103, China; cpummm@163.com (Z.M.); cgyxfys@163.com (G.C.); 3 National Glycoengineering Research Center, Shandong University, Jinan 250012, China; 4 Key Laboratory of Chemical Biology, Ministry of Education, Shandong University, Jinan 250012, China

**Keywords:** variable selection, near-infrared spectroscopy, data binning, normalized mutual information

## Abstract

Variable (wavelength) selection is essential in the multivariate analysis of near-infrared spectra to improve model performance and provide a more straightforward interpretation. This paper proposed a new variable selection method named binning-normalized mutual information (B-NMI) based on information entropy theory. “Data binning” was applied to reduce the effects of minor measurement errors and increase the features of near-infrared spectra. “Normalized mutual information” was employed to calculate the correlation between each wavelength and the reference values. The performance of B-NMI was evaluated by two experimental datasets (ideal ternary solvent mixture dataset, fluidized bed granulation dataset) and two public datasets (gasoline octane dataset, corn protein dataset). Compared with classic methods of backward and interval PLS (BIPLS), variable importance projection (VIP), correlation coefficient (CC), uninformative variables elimination (UVE), and competitive adaptive reweighted sampling (CARS), B-NMI not only selected the most featured wavelengths from the spectra of complex real-world samples but also improved the stability and robustness of variable selection results.

## 1. Introduction

In recent years, near-infrared (NIR) spectroscopy has been widely used in agriculture [1,2], petrochemical engineering [3,4], pharmaceutical [5,6,7], food [8,9], forestry [10], traditional Chinese medicine [11,12,13], environmental [1,14], and biomedical fields [15,16,17,18] due to its rapid, non-invasive, and no-sample-preparation characteristics. And infrared (IR) spectroscopy is often used as a representative example of coordination chemistry analysis [19,20,21,22,23]. Unlike IR spectroscopy, however, NIR spectroscopy is used in conjunction with chemometrics for qualitative or quantitative analysis because the spectral bands are susceptible to complex external factors, making the spectra hard to interpret.

The combination of chemometrics [24,25,26] and spectroscopy regroups several related topics, such as preprocessing methods, variable selection methods, qualitative and quantitative modeling, and experimental design. Generally, the large amount of spectral data leads to the appearance of some noise and irrelevant variables, which makes the predicted properties of the target compounds unreliable. Therefore, some suitable projection or selection techniques have been developed to address these problems.

Projection methods, like partial least squares (PLS) [27] and principal component regression (PCR) [28], typically substitute the original high-dimensional variable space with the low-dimensional space to reduce the impact of collinearity and overlapping bands. However, even with such complex chemometric methods as PLS, the effect of extraneous variables in the spectra cannot be completely eliminated. The influence of data that contain noise or other redundant information may severely corrupt calibration models [29].

In contrast, variable selection methods use algorithms to choose leaner variables that carry information related to the attributes of interest. The variable selection can improve the model stability and interpretability if variables carrying pertinent information are correctly selected [30]. The relevant variables are typically selected using filter-based, extreme value, sequential, exhaustive, and model population analysis search methods [31]. Common variable selection methods include VIP, CC, UVE, CARS, etc.

The variable importance projection is mainly used for variable screening, and the VIP based on partial least squares regression (PLSR) can be used in the case of a small sample size and strong correlation between several independent variables [31]. The variable is considered significant when the mean VIP value and one standard deviation of its bootstrap are greater than 1.0 [32]. The application of the VIP algorithm is pivotal in the creation of the PLS model.

The BIPLS algorithm is similar to the interval PLS model and has been shown to be more precise and reliable than conventional PLS [33]. The basic principle of BIPLS is to divide all variables into a large number of intervals of equal width, assuming that the number of intervals is *n*. And then the PLS models are calculated with each interval left out in a sequence. Among these developed PLS models, the combination with the smallest RMSECV value is selected [33,34].

The CC method is a test correlation calculation of the absorbance vector in the spectral matrix corresponding to each wavelength and the concentration vector under the density matrix component in order to obtain a wavelength correlation coefficient map [35]. And the model with a wavelength correlation coefficient greater than a certain threshold is selected. CC is a common approach for performing band selection and is more frequently used in building NIR prediction models [36,37,38].

The UVE method is an algorithm based on the analysis of the PLS regression coefficients β for eliminating those variables that do not provide information. Based on the criterion judgment of β coefficients, the experimental variables with lower importance are eliminated, and then the model is built. Finally, the method has been proven to improve prediction ability [39].

The CARS method utilizes a combination of Monte Carlo sampling and regression coefficients from the PLS model to select feature variables [40]. In the CARS algorithm, the points in the PLS model with larger absolute weights of regression coefficients are kept as a new subset using adaptive reweighted sampling (ARS). The PLS model is developed with the new subset, which removes the points with smaller weights. The wavelength in the subset with the smallest root mean square error of the cross-validation (RMSECV) of the PLS model is selected as the feature wavelength after multiple calculations.

Conventional variable selection methods are mainly based on the theory of projection or regression coefficients. A major drawback of all these methods is that they are not invariant under the transformation of variables, which may modify the results due to small changes in the variables. And they are sensitive to noise or outlier data in training data and it is hard to detect redundant features. However, the information measures investigate the amount of information or the uncertainty of a feature for variable selection [41]. The central idea of information theory is that the “information value” of a communication message depends on the degree of surprise of the message content, which is widely used in feature selection [42,43,44]. Mutual information is a well-known concept in information theory, reflecting the degree of linear or nonlinear dependence between the variables [45,46].

In this study, a variable selection method based on the information entropy of “binning-normalized mutual information” was proposed for the first time for multi-component spectral calibration. The combination of the two methods enables the maximum calculation of the relationship between the spectral variables and the reference value, including linear and non-linear relations [47]. The irrelevant background information in the spectra was effectively removed, which was particularly prominent in complex real-world samples. The feasibility and accuracy of the B-NMI approach were shown by the statistical parameters of the prediction model on four different datasets, including the ideal ternary solvent mixture dataset, fluidized bed granulation dataset, gasoline octane dataset (public data), and corn protein dataset (public data). Furthermore, to illustrate its superiority, the B-NMI method was compared with five classical variable selection methods (BIPLS, VIP, CC, UVE, CARS).

## 2. Results and Discussion

### 2.1. Model Analysis of Ideal Ternary Solvent Mixture Dataset

The BIPLS, VIP, CC, UVE, and CARS were compared in this study to evaluate the performance of the B-NMI method. It is crucial to select the appropriate number of LVs in the PLSR model, as too many or too few LVs may cause overfitting or underfitting problems in the predicted model. In this study, the number of LVs in the model was determined by the leave-one-out cross-validation method. The smallest RMSECV point (or inflection point) was considered to be the optimal number of LVs. In the solvent mixture dataset, the three replicate spectra were averaged before data processing. The by-default pretreatment technique, mean centering, was used as the default pre-processing method for PLSR analysis to preprocess spectral data. Other preprocessing techniques, such as the first derivative or standard normal variate, mainly used to eliminate the baseline caused by solid scattering, were not tested in this liquid dataset.

**Figure 1:**
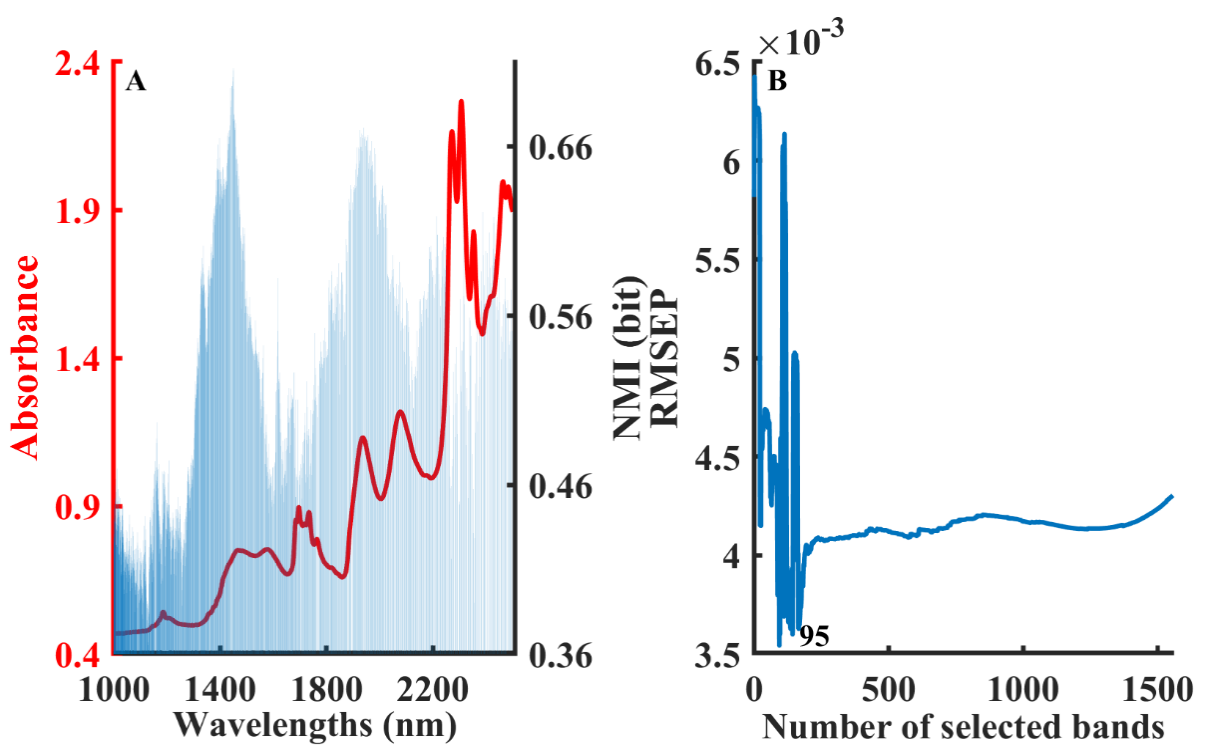
The procedure of the B-NMI method for moisture content in solvent mixture dataset: NMI values distribution in different wavelengths (**A**), variation in RMSEP by developing model with cumulative wavelengths in the order of NMI values (**B**).

Figure 1A shows the distribution of the NMI values at different wavelengths under the optimal modeling results after calculating with a different number of binned box iterations. The left *y*-axis represents the absorbance (red line) and the right *y*-axis represents the NMI value between each variable and water content (blue bar). Figure 1A intuitively displays significant differences in the NMI values at different wavelengths. The NMI value mainly reflects the relevance between two variables, which can be considered as a basis for judging the importance of variables to the PLSR model. Figure 1B shows the change in the RMSEP of the water content PLS model developed by a sequential accumulation of wavelengths in the order of NMI values from largest to smallest. The RMSEP decreased rapidly in the first stage as the variables with larger NMI values were added to the model, and then increased in the second stage. The RMSEP reached its minimum value when 95 variables were selected for modeling.

**Figure 2:**
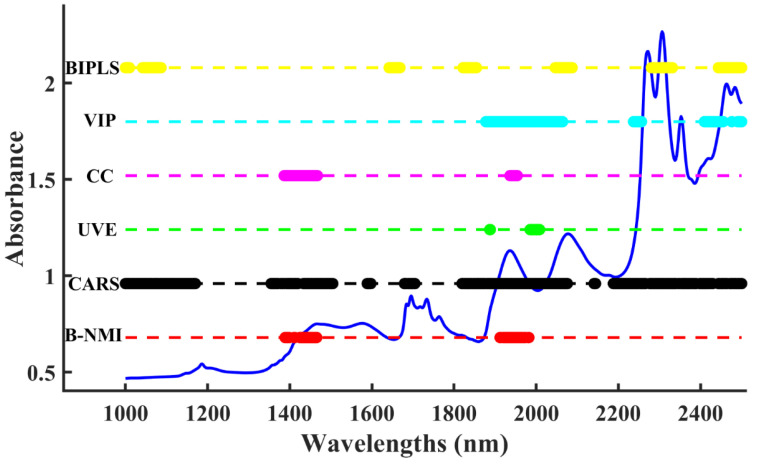
Visual comparison of selected variables for moisture content using different algorithms in the solvent mixture dataset.

The B-NMI was compared with five widely used variable selection strategies. The selected important wavelengths for water content are shown in Figure 2. As each variable selection algorithm works differently, the water content variable selected varies greatly. In general, water bands in the near-infrared region around 1450 and 1940 nm were used to determine water content [48]. The dominant spectral region for all methods was 1300–1600 nm or 1900–2200 nm, which can be attributed to the first tone of the O-H stretching mode and the combination of the O-H bond [40], respectively. The bands selected for B-NMI, UVE, and CC were highly correlated with water absorption. In contrast, the BIPLS, CARS, and VIP selected many bands that are not relevant to water.

Table 1 summarizes the predicted results of the PLSR models developed using different selection methods for water content. All variable selection methods outperformed the full-spectral PLS. Moreover, the performances of B-NMI, UVE, and CC were better than those of BIPLS, VIP, and CARS due to their highly correlated bands with water, which proved the feasibility of B-NMI in selecting correlation bands with water. However, the performance of UVE was better than that of B-NMI in the simple ternary solvent mixture. In a simple system with low background interference noise, complex processing methods like B-NMI may not be necessary to effectively extract feature bands. As a result, the superiority of B-NMI may not be reflected in such a simple solution system.

### 2.2. Model Analysis of Fluidized Bed Granulation Dataset

**Figure 3:**
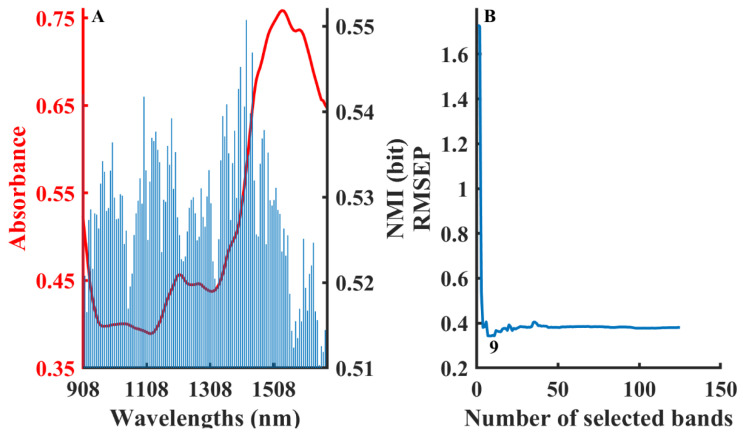
The procedure of the B-NMI method for moisture content in fluidized bed granulation dataset: NMI values distribution in different wavelengths (**A**), variation in RMSEP by developing model with cumulative wavelengths in the order of NMI values (**B**).

During fluidized bed granulation, moisture as a critical quality attribute affects the subsequent processing and drug stability [49]. Too much moisture may lead to tablet adhesion and aggregation, while too little moisture may lead to delamination or fragility of the tablets [50,51]. Figure 3 shows the procedure of the B-NMI method. The high NMI values were mainly distributed in the range of 1300–1500 nm (Figure 3A), which corresponds to water absorption. Figure 3B shows that the optimal PLS model was developed using nine wavelengths with high NMI values.

**Figure 4:**
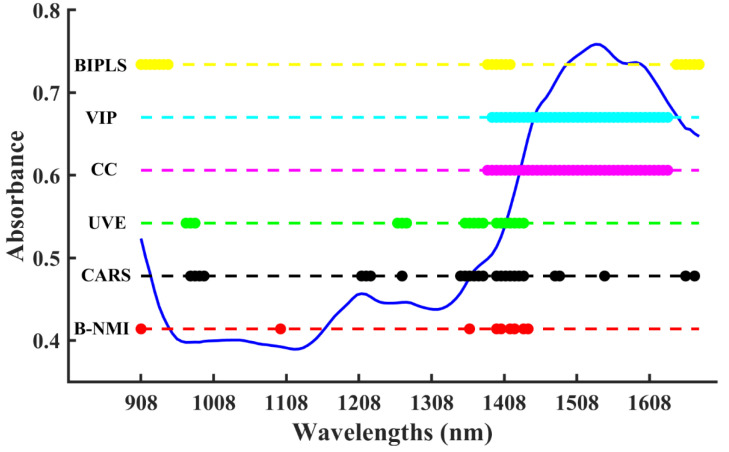
Visual comparison of selected variables for moisture content using different algorithms in the fluidized bed granulation dataset.

The visual plot and predicted results of all variable selection methods for water content in fluidized bed granulation are shown in Figure 4 and Table 2, respectively. During the granulation process, the material was in a dynamic flow state. Moreover, external conditions such as temperature, humidity, and pressure were constantly fluctuating, leading to a complex background of disturbances in the NIR spectra. The B-NMI method can effectively remove the noise and select the bands around 1450 nm, which corresponds to the first overtone of the O-H stretching mode and reflects the change in the water. Other selection methods, such as VIP and CC, selected uncorrelated wavelengths of water around 1500–1600 nm, which represents the characteristic band of the adhesive HPMC, presenting a worse performance. In summary, the B-NMI method was effective in selecting the most informative bands in a complex background, leading to a better performance compared to other selection methods. It enabled the accurate identification of changes in water during the granulation process, even in the presence of external disturbances.

Moreover, the model performance of all selection methods after SNV preprocessing was also compared to prove the robustness of the B-NMI method (Table S1, Supplementary Materials). The SNV was mainly chosen to remove the baseline offset and slope caused by a variety of physical factors, such as particle size and optical patches. The SNV method did not improve the predictive capability of the model compared to the raw spectra. However, B-NMI still presented the best prediction results compared to other band selection methods.

### 2.3. Model Analysis of Gasoline Octane Dataset

**Figure 5:**
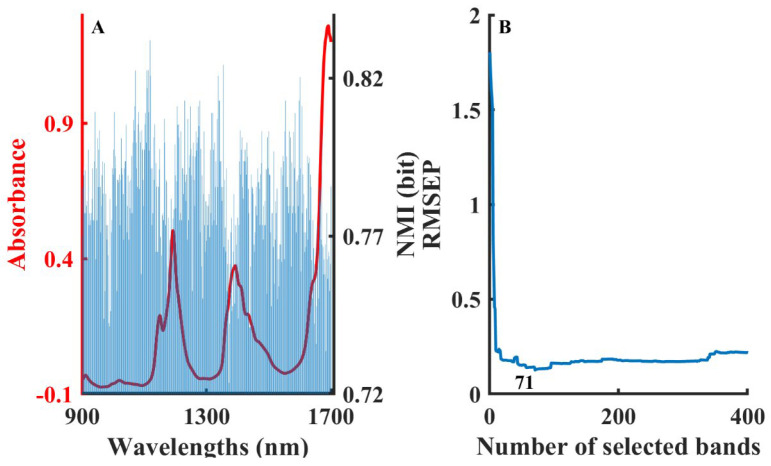
The procedure of the B-NMI method for octane content in gasoline dataset: NMI values distribution in different wavelengths (**A**), variation in RMSEP by developing model with cumulative wavelengths in the order of NMI values (**B**).

One of the most vital indicators of gasoline is the octane number (ON), which is an empirical indicator for evaluating the strength of gasoline against striking [52]. The composition of gasoline is complex. The main components of gasoline are C5~C12 aliphatic hydrocarbons and naphthene, with some aromatics. It can also be seen from the NMI distribution plot in Figure 5A that the high NMI values were distributed throughout the band. Figure 5B shows that 71 wavelengths with high NMI values needed to be used to build the best PLS prediction model.

**Figure 6:**
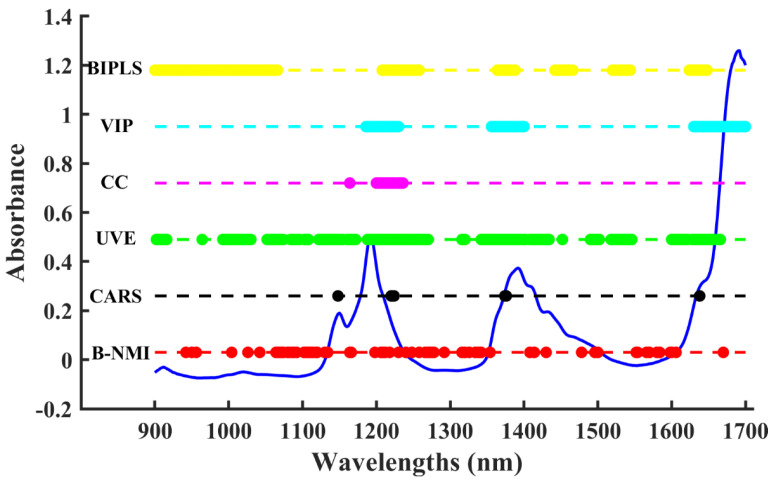
Visual comparison of selected variables for octane content using different algorithms in the gasoline dataset.

The visual plot (Figure 6) of variable selection displays the selected wavelengths of octane mainly located at the following sub-ranges: 1550–1600 nm range involving the first harmonic (2ν) and a combination (ν + 2δ) of the –CH’s stretching and deformation vibration; 1200–1400 nm, including the (2ν + δ) bands; and 1000–1200 nm, including the (3ν) and [2(ν + δ)] bands [53]. Table 3 summarizes the predicted results of the PLSR models developed using different selection methods for octane. The performance of B-NMI was significantly better than other methods, which proved the superiority of B-NMI in selecting correlation bands in complex samples. Other methods either selected too many irrelevant variables (UVE) or selected few relevant variables (VIP, CC), all showing a poor predictive performance. In addition, there was a significant improvement in B-NMI predictions compared to the octane values predicted by other researchers [54].

### 2.4. Model Analysis of Corn Protein Dataset

**Figure 7:**
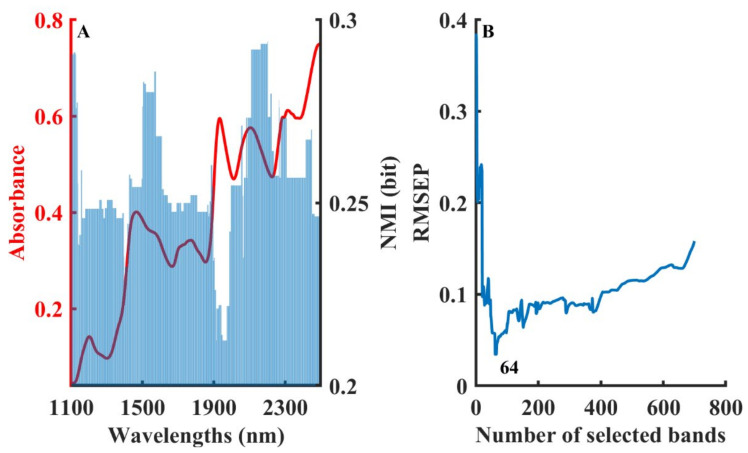
The procedure of the B-NMI method for protein content in corn dataset: NMI values distribution in different wavelengths (**A**), variation in RMSEP by developing model with cumulative wavelengths in the order of NMI values (**B**).

Corn is a popular staple food in many countries around the world, and protein content is one of the vital indicators in determining the nutritional value of corn. Moreover, there have been many research methods that have tested public corn data [55,56], while corn protein data seem to be more difficult to predict. The signal of protein may be masked by other major components of corn, such as carbohydrates, fat, water, and crude fiber. Therefore, the superiority of the B-NMI method was further tested with a complex corn dataset. Figure 7A shows that there were two distributions of high NMI values in the ranges of 1500–1600 nm and 2100–2300 nm, which correspond to the absorption of protein [17]. Figure 7B shows that the optimal PLS model was developed using 64 wavelengths with high NMI values.

**Figure 8:**
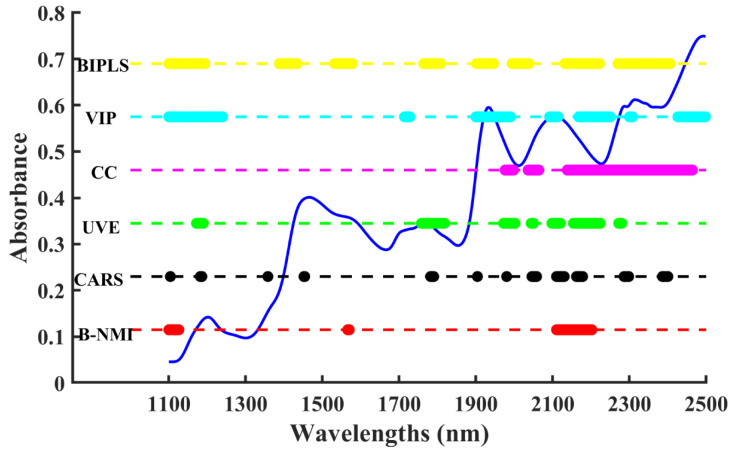
Visual comparison of selected variables for protein content using different algorithms in the corn dataset.

Figure 8 displays the selected wavelengths of the corn protein dataset, mainly located in the range of 2100–2200 nm, which were assigned as the amide A-amide II combination and the amide B-amide II combination bands [57]. And the 2000–2500 nm region was reported to be useful for protein structural characterization and quantification [58,59], which proved the accuracy of the B-NMI method in selecting variables. The B-NMI method not only effectively identifies and eliminates irrelevant variables but also removes redundant variables to extract the most prominent variables.

Table 4 summarizes the predicted results of different selection methods, which show that the B-NMI prediction performance was significantly superior to other methods. Furthermore, a comparison with the corn protein predictions made by other researchers [60] demonstrates a significant enhancement in B-NMI. The SNV was also chosen to eliminate the effect of scattering in solid samples (Table S2, Supplementary Materials). The SNV method enhances the predictive capability of the full PLSR model compared to the original raw spectra. However, the preprocessing methods combined with variable selection methods reduced the model performance. The main reason may be that the SNV removes the baseline while also eliminating some spectral information.

At last, an F-test was performed to compare the statistical significance of the RMSEP values of the B-NMI method with other variable selection methods, where a confidence level of 95% was adopted [61]. The results are displayed in Table 5. For the simple solvent mixture dataset, the F-test showed that the prediction results of the B-NMI method were equivalent to those of the selection methods, and the enhancement effect was not obvious. However, it can be seen that *p*-values for granulation, gasoline octane, and corn protein were obviously less than 0.05, which means that the B-NMI method was significantly different from those selection methods in modeling prediction. The above results show that the effectiveness of the B-NMI method in selecting characteristic bands may not be as apparent in simpler systems, but it becomes increasingly prominent in more complex systems. This suggests that the B-NMI method could be particularly useful for tasks that involve the analysis of complex data, such as real-world sample processing, where identifying relevant features is crucial for accurate analysis.

## 3. Theory and Algorithms

**Figure 9:**
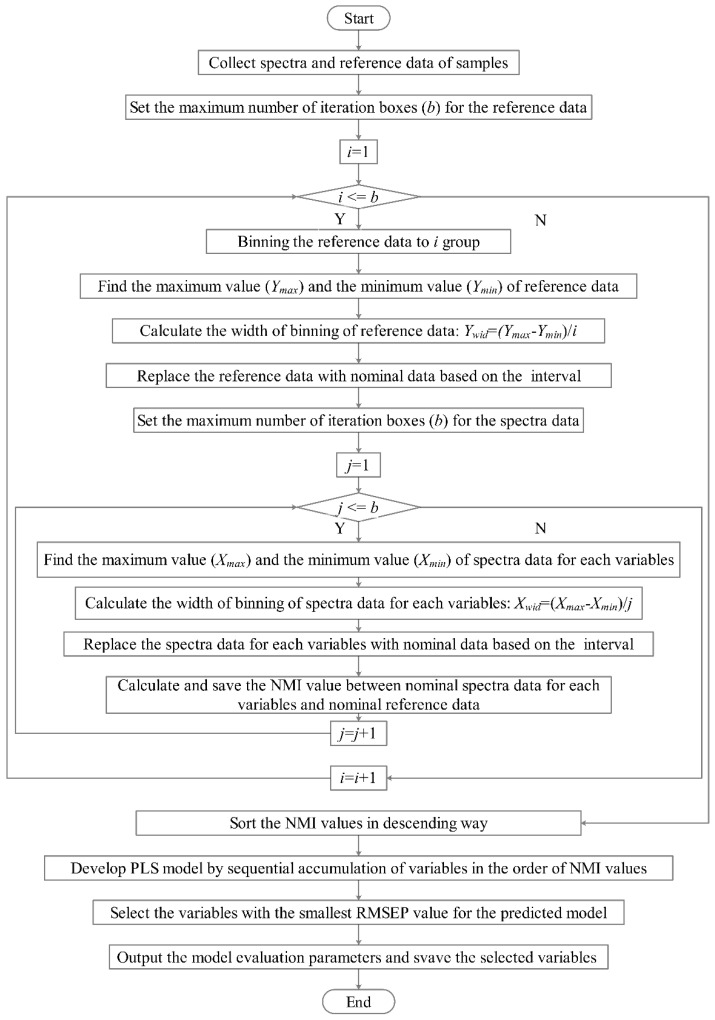
Flow chart of the B-NMI algorithm.

Matlab 2018a (Mathworks, Natick, MA, USA) and Pycharm 2021 (JetBrains, Prague, Czech Republic) were adopted for data processing. The flowchart of the B-NMI procedure is illustrated in Figure 9. It can be summarized in the following steps:

A spectra dataset matrix X(*m* × *n*) contains *m* samples in rows and *n* variables in columns. A reference dataset matrix Y(*m* × 1) contains *m* samples in rows.

Pre-processing the original data (spectra and reference) with the data binning (equal intervals) method (see Section 3.1).

Calculating the normalized mutual information (NMI) between spectra data for each variable and reference data (see Section 3.3).

Sorting the NMI values in descending order.

Developing the PLS model by sequentially adding variables in the order of NMI values.

Selecting the variables with the smallest root mean square error of prediction (RMSEP) value.

In this paper, this is the first time that information extropy theory is applied to the processing of spectra, which will be an alternative method with an excellent performance. The novelty of this paper lies in several key areas. Firstly, the use of data binning helps to reduce noise and improve accuracy in the estimation of NMI, which leads to more precise band selection. This is particularly relevant in the context of near-infrared band selection, where the noise level can be high in complex real-world samples. Secondly, the use of NMI as a measure of the relationship between variables allows for the identification of both strong and weak relationships, leading to the selection of more informative and relevant bands. NMI is a more robust and flexible measure than traditional methods, such as correlation coefficient, as it does not assume linear relationships between variables and can detect non-linear correlations. Thirdly, the sequential addition of variables based on NMI values allows for a more efficient and targeted selection process. This approach ensures that the most relevant bands are selected early in the process, leading to an improved model performance. Finally, the use of NMI also allows for the selection of bands that are more independent and less redundant, which can further improve the performance of the model. By selecting the most informative and independent bands, the model can better capture the underlying relationships between the variables.

### 3.1. Data Binning

Data binning is a data preprocessing technique used to reduce the effects of observation errors. In statistical analysis, data binning is used to convert or partition continuous variables into discretized or nominal variables to enhance the characteristics of variables. Typically, the data are discretized into partitions of B equal lengths/width (equal intervals) or B% of the total data (equal frequencies) [62]. In this paper, the spectra and reference data are processed using the data binning (equal intervals) method. This consists of four stages:

Determining the number of the box ($B_{number}$), which is generally twice the number of samples; note that the $B_{number}$ here is not the number of bins for subsequent modeling, but the number of bins for the maximum iteration.

Calculating the width of the box,

(1) $$B_{wid}=\frac{{(D}_{max}-D_{min})}{B_{number}}$$

where $D_{max}$ and $D_{min}$ represent the maximum and minimum values in the data column, respectively. The interval boundary values are $D_{min}+B_{wid}$, $D_{min}+2B_{wid}$, …, $D_{min}+(B_{number}-1)B_{wid}$.

Replacing the original data with nominal data that fall into a given small interval based on the value $B_{wid}$.

Calculating the results of all data bins by exhaustive enumeration.

### 3.2. Mutual Information (MI)

MI is a good method for analyzing the correlation between two variables (spectra data and reference data). For two variables $X_{i}$ and $Y_{j}$, MI is the measure of the interdependence between these two variables (absorbance values for each wavelength and reference data after processing by binning method). It is defined as

(2) $$MI(X_{i},Y_{j})=H(X_{i})-H(X_{i}|Y_{j})$$

where $H(X_{i})$ is the marginal entropy of absorbance variable $X_{i}$, defined as

(3) $$H\left(X_{i}\right)=-\sum_{i}p(x_{i})\log\;p(x_{i})$$

and $H(X_{i}|Y_{j})$ is the conditional entropy:

(4) $$H\left(X_{i},|,Y_{j}\right)=-\sum_{j}p(y_{j})\sum_{i}p(x_{i}|y_{j})\log\;p(x_{i}|y_{j})$$

where $p(y_{j})$ is the probability of reference $y_{j}$ and $p(x_{i}|y_{j})$ is the posterior probability of absorbance $x_{i}$ given reference $y_{j}$.

However, mutual information tends to increase its value with an increase in the number of values of $X_{i}$ and/or $Y_{j}$, which means that MI is biased to the cardinality features. Therefore, MI has to be normalized with the entropies of the features to eliminate such bias [63]:

(5) $$MI\left(X,Y\right)=\sum_{i=1}^{X}\;\sum_{j=1}^{Y}\;p_{i,j}\log\;(\frac{P_{i,j}}{P_{i}\times {\;P}_{j}})$$

### 3.3. Normalized Mutual Information (NMI)

This information-based nonlinear measure, known as *symmetrical uncertainty*, is the normalized version of MI. It rescales the MI score into a numerical value between 0 and 1.

Now, notice that, if $X_{i}$ and $Y_{j}$ are independent, then $NMI(X_{i},Y_{j})\;$= 0; and (ii) if $X_{i}$ and $Y_{j}$ are fully correlated, then $NMI(X_{i},Y_{j})$ = 1. Therefore, NMI values are in the range [0, 1]. NMI can measure the correlation between two variables and is often used in variable selection methods [64]. This equation has two variables, $X_{i}$ and $Y_{j},$ and is determined as follows:

(6) $$\mathrm{NMI}\left(X_{i},Y_{j}\right)=\frac{2\times MI\left(X_{i},Y_{j}\right)}{H\left(X_{i}\right)+H\left(Y_{j}\right)}=2\times \frac{H(X_{i})-H(X_{i}|Y_{j})}{H(X_{i})+H(Y_{j})}$$

### 3.4. Evaluation Criteria

The criteria used to evaluate the performance of the model include determination coefficient R^2^, root mean square error of validation (RMSEP), and ratio of performance deviation (RPD). The closer R^2^ is to 1, the better the regression or prediction will be. Lower values of RMSEP indicate greater accuracy in predicting the target component. RPD is calculated as the ratio of the standard deviation of the reference values to the RMSEP. Higher values of RPD indicate a greater precision and reliability of the model.

The calculation formulas are as follows:

(7) $$R^{2}=1-\frac{\sum_{i=1}^{n}{\left(y_{i,\;actual}-y_{i,\;predicted}\right)}^{2}}{\sum_{i=1}^{m}{\left(y_{i,\;actual}-{\bar{y}}_{i,\;actual}\right)}^{2}}$$

(8) $$RMSEP=\sqrt{\frac{\sum_{i=1}^{n}{\left(y_{i,\;actual}-y_{i,\;predicted}\right)}^{2}}{m-1}}$$

(9) $$PD=\frac{{SD}_{actual}}{RMSEP}$$

where $y_{i,\;actual}$ is the reference value of the ith sample, $y_{i,\;predicted}$ is the predicted value of the ith sample, ${\bar{y}}_{i,\;actual}$ is the mean of the reference values, and m is the number of samples. Typically, a satisfactory model will have a high *R*^2^ and RPD and low *RMSEP*.

## 4. Datasets

### 4.1. Ideal Ternary Solvent Mixture Dataset

The ideal ternary solvent mixtures consisting of water, ethanol, and acetic acid were prepared. The NIR spectra were collected from 10,000 to 4000 cm^−1^ with a resolution of 8 cm^−1^ (1557 points) in transmission mode using the Antaris II Fourier transform near-infrared spectrophotometer (Thermo Fisher Scientific, Waltham, MA, USA). A total of 156 spectra (52 mixtures and 3 replicate measurements) were collected for the model development. The calibration set included six concentrations of water (2%, 4%, 6%, 8%, 10%, and 12%), and the corresponding six concentrations of acetic acid range (1%, 3%, 5%, 7%, 9%, and 11%), for a total of thirty-six samples. The validation set included eight concentrations of water (3%, 4%, 5%, 6%, 7%, 8%, 9%, and 10%). Each concentration was measured twice in duplicate for a total of 16 samples. The corresponding concentrations of acetic acid and ethanol were randomly distributed to challenge the robustness of the calibration model. The total volume of all solutions was kept constant. The proportion of water was considered as a reference value.

### 4.2. Fluidized Bed Granulation Dataset

The granulation dataset was created using a portable NIR spectrometer (Micro NIR PAT-U) combined with a fiber optic probe (VIAVI, Chandler, AZ, USA), which used a nominal wavelength range of 908.1–1676.2 nm with a wavelength separation of approximately 6 nm (125 points). The spectra were gathered every 6 s in real time during fluidized bed granulation. In the model development stage, 15 samples (approximately 10 g per sample) were thieved from each batch, yielding 135 samples in 9 batches. Batches 1–5 were calibration sets, and batches 6–9 were validation sets. The moisture content of thieved samples was determined by the drying to constant weight method using a halogen moisture analyzer (XY-102MW, Xinyun, Shanghai, China).

### 4.3. Gasoline Octane Dataset

The gasoline data were from the appendix of the published article [65]. This dataset contained 60 gasoline samples with specified octane values that were measured using diffuse reflectance from 900 to 1700 nm at 2 nm intervals (401 points). These 60 samples were split into a calibration set (45 samples) and a validation set (15 samples) by the KS algorithm.

### 4.4. Corn Protein Dataset

The corn dataset is publicly available and can be downloaded from a website (https://eigenvector.com/resources/data-sets/#corn-sec, accessed on 1 December 2022). This dataset contained 80 samples measured by three different NIR spectrometers (m5, mp5, and mp6) in the spectral range of 1100–2498 nm at 2 nm intervals (700 points). The corresponding reference values (moisture, oil, protein, and starch) of these samples obtained using laboratory analysis are also available. In the present study, only the protein content of the dataset measured by an m5 spectrometer was considered. These 80 samples were split into a calibration set (60 samples) and a validation set (20 samples) by the KS algorithm. Kennard–Stone (KS) is a technique designed to achieve uniform coverage across a multidimensional space by maximizing the Euclidean distances between the instrumental response vectors (x) of the selected samples [66]. Table 6 shows the descriptive statistics for the solvent mixture, granulation, gasoline octane, and corn protein data.

## 5. Conclusions

This paper proposed a novel variable selection method based on information entropy theory that combined the “Data binning” algorithm and the “Normalized mutual information” method, named B-NMI. Four datasets, including two experimental datasets and two public datasets, were used to demonstrate the performance of the novel proposed B-NMI method. And the B-NMI method was also compared with five different wavelength selection methods (BIPLS, VIP, CC, UVE, CARS) to demonstrate its superiority. The B-NMI method showed a better predictive ability in these datasets due to effective feature extraction and highly relevant model development, especially in processing complex real-world samples. The B-NMI methods can not only identify and eliminate irrelevant variables effectively but also remove the redundant ones by evaluating all probability results calculated by an exhaustive search. The present study demonstrates the feasibility and effectiveness of the B-NMI method, which will be an effective and prospective tool for determining target components in complex samples in practice. Furthermore, there exist captivating opportunities for leveraging information entropy in various domains, such as preprocessing method screening, outlier determination, cluster analysis, and data fusion. The inherent capability of information entropy to effectively extract valuable information makes it an indispensable tool in these applications. Additionally, the integration of information entropy with deep learning methods holds immense promise, opening up new avenues for advanced data analysis and decision making.

## Figures and Tables

**Table 1:** The results of PLSR model in different variable selection methods for the solvent mixture dataset.

Models	R^2^_C_	R^2^_P_	RMSEC	RMSECV	RMSEP	RPD	Bias	Number of Variables	LVs
FULL-PLSR	0.986	0.965	0.00401	0.00470	0.00430	5.499	−0.003	1557	3
VIP-PLSR	0.985	0.970	0.00414	0.00454	0.00399	5.927	−0.003	164	3
CC-PLSR	0.985	0.971	0.00412	0.00456	0.00387	6.107	−0.003	116	3
**UVE-PLSR**	**0.985**	**0.978**	**0.00424**	**0.00457**	**0.00340**	**6.950**	**−0.002**	**18**	**2**
CARS-PLSR	0.986	0.966	0.00403	0.00459	0.00419	5.642	−0.003	866	3
BIPLS	0.985	0.966	0.00420	0.00435	0.00420	5.572	−0.004	259	2
B-NMI-PLSR	0.985	0.976	0.00412	0.00476	0.00354	6.679	−0.002	95	3

Bold indicated the optimal variable selection method.

**Table 2:** The results of PLSR model in different variable selection methods for the fluidized bed granulation dataset.

Models	R^2^_C_	R^2^_P_	RMSEC	RMSECV	RMSEP	RPD	Bias	Number of Variables	LVs
FULL-PLSR	0.976	0.966	0.312	0.322	0.380	5.438	−0.040	125	5
VIP-PLSR	0.974	0.965	0.325	0.333	0.387	5.345	−0.061	40	4
CC- PLSR	0.977	0.968	0.308	0.321	0.367	5.626	−0.025	41	5
UVE-PLSR	0.975	0.970	0.318	0.326	0.356	5.813	−0.017	18	5
CARS-PLSR	0.978	0.968	0.303	0.312	0.370	5.581	−0.036	26	5
BIPLS	0.978	0.969	0.303	0.296	0.362	5.709	−0.097	19	5
**B-NMI-PLSR**	**0.977**	**0.972**	**0.308**	**0.316**	**0.343**	**6.027**	**0.021**	**9**	**5**

Bold indicated the optimal variable selection method.

**Table 3:** The results of PLSR model in different variable selection methods for the gasoline octane dataset.

Models	R^2^_C_	R^2^_P_	RMSEC	RMSECV	RMSEP	RPD	Bias	Number of Variables	LVs
FULL-PLSR	0.990	0.987	0.150	0.252	0.180	9.013	0.000	401	6
VIP-PLSR	0.988	0.986	0.165	0.260	0.184	8.808	−0.001	82	6
CC-PLSR	0.987	0.989	0.171	0.288	0.165	9.873	0.005	20	7
UVE-PLSR	0.987	0.987	0.171	0.216	0.180	9.015	0.001	217	4
CARS-PLSR	0.989	0.978	0.159	0.187	0.240	6.950	0.041	7	4
BIPLS	0.978	0.992	0.218	0.221	0.144	11.313	0.053	162	3
**B-NMI-PLSR**	**0.981**	**0.994**	**0.205**	**0.255**	**0.126**	**12.905**	**0.016**	**71**	**5**

Bold indicated the optimal variable selection method.

**Table 4:** The results of PLSR model in different variable selection methods for the corn protein dataset.

Models	R^2^_C_	R^2^_P_	RMSEC	RMSECV	RMSEP	RPD	Bias	Number of Variables	LVs
FULL-PLSR	0.958	0.879	0.106	0.151	0.146	2.951	0.028	700	8
VIP-PLSR	0.925	0.903	0.142	0.179	0.131	3.293	0.025	221	7
CC- PLSR	0.952	0.967	0.113	0.144	0.076	5.635	−0.014	191	8
UVE-PLSR	0.971	0.985	0.088	0.117	0.051	8.411	−0.005	114	7
CARS-PLSR	0.979	0.951	0.074	0.097	0.092	4.651	0.003	51	8
BIPLS	0.986	0.992	0.062	0.147	0.038	11.284	0.028	280	6
**B-NMI-PLSR**	**0.987**	**0.993**	**0.059**	**0.077**	**0.035**	**12.446**	**−0.003**	**64**	**7**

Bold indicated the optimal variable selection method.

**Table 5:** Comparisons of the predictive ability of three methods using F-test for a confidence level of 95%.

Methods	Datasets			
	Solvent Mixture	Granulation	Gasoline Octane	Corn Protein
B-NMI vs. VIP *p*-values	0.494	0.000	0.038	0.000
B-NMI vs. CC *p*-values	0.732	0.000	0.915	0.000
B-NMI vs. UVE *p*-values	0.947	0.028	0.042	0.000
B-NMI vs. CARS *p*-values	0.324	0.000	0.002	0.000
B-NMI vs. BIPLS *p*-values	0.026	0.000	0.002	0.000

**Table 6:** Descriptive statistics of the four datasets.

Data	N	Calibration Set		N	Validation Set	
		Mean ± SD	Range		Mean ± SD	Range
Solvent mixture	36	0.07 ± 0.04	0.02–0.12	16	0.07 ± 0.02	0.03–0.10
Granulation	75	5.62 ± 2.03	3.27–11.83	60	5.88 ± 2.07	3.02–10.94
Gasoline octane	45	87.28 ± 1.50	83.40–89.60	15	86.87 ± 1.63	84.50–88.90
Corn protein	60	8.67 ± 0.52	7.65–9.71	20	8.68 ± 0.43	7.79–9.44

## Data Availability

The data is contained within the article.

## Supplementary Materials

The following supporting information can be downloaded at: https://www.mdpi.com/article/10.3390/molecules28155672/s1, Table S1: The results of PLSR model after SNV preprocessing in different variable selection methods for the fluidized bed granulation dataset. Table S2: The results of PLSR model after SNV preprocessing in different variable selection methods for the corn protein dataset.
