# Supplementary material for: A Novel Variable Selection Method Based on Binning-Normalized Mutual Information for Multivariate Calibration
Source: Molecules. 2023 Jul 26;28(15):5672. doi: 10.3390/molecules28155672 (PMC10419756; doi:10.3390/molecules28155672)
Supplement: Supplementary file 1 [file molecules-28-05672-s001.zip › molecules-2418820-supplementary.pdf]

Supporting Information

A novel variable selection method based on binning-normalized mutual information for multivariate calibration

Liang Zhong[a,†], Ruiqi Huang[a,†], Lele Gao[a] , Jianan Yue[a], Bing Zhao[a], Lei Nie[a], Lian Li[a], Aoli Wu[a], Kefan Zhang[a], Zhaoqing Meng[d], Guiyun Cao[d], Hui Zhang[a,b,*], Hengchang Zang[a,b,c,*]

[a] NMPA Key Laboratory for Technology Research and Evaluation of Drug Products, School of Pharmaceutical Sciences, Cheeloo College of Medicine, Shandong University, Jinan, 250012, Shandong, China

[b] National Glycoengineering Research Center, Shandong University, Jinan 250012, Shandong, China

[c] Key Laboratory of Chemical Biology (Ministry of Education), Shandong University, Jinan, 250012, China

[d] Shandong Hongjitang Pharmaceutical Group Co. Ltd., Jinan 250103, China

* Correspondence: zanghcw@126.com; Tel.: +86 531 88380268

*Hui Zhang and Hengchang Zang are corresponding authors.

† These authors contributed equally to this work and should be regarded as co-first authors.

Table S1. The results of PLSR model after SNV preprocessing in different variable selection methods for the fluidized bed granulation dataset.

Models	R^2_C	R^2_P	RMSEC	RMSECV	RMSEP	RPD	Bias	Number of variables	LVs
FULL-PLSR	0.975	0.964	0.321	0.334	0.390	5.304	-0.055	125	5
VIP-PLSR	0.951	0.956	0.451	0.460	0.430	4.805	-0.090	30	3
CC- PLSR	0.975	0.965	0.321	0.334	0.388	5.325	-0.051	123	5
UVE-PLSR	0.975	0.966	0.322	0.335	0.382	5.405	-0.022	72	5
CARS-PLSR	0.972	0.963	0.341	0.349	0.396	5.219	-0.055	5	4
BIPLS	0.976	0.967	0.313	0.313	0.377	5.483	-0.127	88	5
**B-NMI-PLSR**	**0.972**	**0.967**	**0.337**	**0.349**	**0.374**	**5.531**	**0.044**	**49**	**5**

Table S2. The results of PLSR model after SNV preprocessing in different variable selection methods for the corn protein dataset.

Models	R^2_C	R^2_P	RMSEC	RMSECV	RMSEP	RPD	Bias	Number of variables	LVs
FULL-PLSR	0.954	0.915	0.111	0.144	0.122	3.517	-0.027	700	7
VIP-PLSR	0.933	0.849	0.134	0.167	0.163	2.644	-0.027	256	8
CC- PLSR	0.942	0.946	0.125	0.167	0.097	4.420	-0.031	126	8
UVE-PLSR	0.963	0.942	0.099	0.146	0.101	4.246	-0.011	287	7
CARS-PLSR	0.974	0.919	0.083	0.103	0.119	3.604	-0.005	21	8
BIPLS	0.968	0.950	0.093	0.110	0.093	4.604	-0.002	163	6
**B-NMI-PLSR**	**0.941**	**0.972**	**0.126**	**0.152**	**0.070**	**6.147**	**-0.009**	**37**	**7**
